# Using automated syllable counting to detect missing information in speech transcripts from clinical settings

**DOI:** 10.1016/j.psychres.2022.114712

**Published:** 2022-07-05

**Authors:** Marama Diaz-Asper, Terje B. Holmlund, Chelsea Chandler, Catherine Diaz-Asper, Peter W. Foltz, Alex S. Cohen, Brita Elvevåg

**Affiliations:** a Washington International School, Washington DC, United States; b Department of Clinical Medicine, University of Tromsø - The Arctic University of Norway, Tromsø, Norway; c Department of Computer Science, University of Colorado Boulder, CO, United States; d Department of Psychology, Marymount University, Arlington, VA, United States; e Institute of Cognitive Science, University of Colorado Boulder, CO, United States; f Department of Psychology, Louisiana State University, LA, United States; g Norwegian Center for eHealth Research, University Hospital of North Norway, Tromsø, Norway

**Keywords:** Automatic speech recognition, Word error rate, Syllables

## Abstract

Speech rate and quantity reflect clinical state; thus automated transcription holds potential clinical applications. We describe two datasets where recording quality and speaker characteristics affected transcription accuracy. Transcripts of low-quality recordings omitted significant portions of speech. An automated syllable counter estimated actual speech output and quantified the amount of missing information. The efficacy of this method differed by audio quality: the correlation between missing syllables and word error rate was only significant when quality was low. Automatically counting syllables could be useful to measure and flag transcription omissions in clinical contexts where speaker characteristics and recording quality are problematic.

## 1. Introduction

Speech rate and quantity can be important proxies for mental and cognitive health. Indeed, a patient’s verbal self presentation forms a critical component of the clinical evaluation, and changes in speech rate are associated with cognitive decline in the elderly (Szatloczki et al., 2015; Themistocleous et al., 2020) and mental state changes in serious mental illness (SMI) (Çokal et al., 2019; Parola et al., 2020; Rapcan et al., 2010). Therefore, how many words a patient utters (word count) can be a valuable diagnostic tool in the clinical setting. As speech analysis technologies become integrated into clinical tools, automated speech recognition (ASR) can be leveraged to automatically assay clinical signs such as the speed and amount of words uttered per unit of time or per prompt (e.g., a question or a task).

While ASR shows great promise in the clinical setting, an accurate and complete ASR transcription is dependent on many factors, including speaker volume, articulation and accent, background noise, distance of the microphone from the speaker, limited audio frequency range in telephone transmissions as well as the type of recording equipment used (Bhattacharjee et al., 2016; Ulasik et al., 2020). Therefore, if ASR tools are to be implemented in clinical settings, it is imperative that their output is robust and valid when the capture of audio data is less than optimal, as the consequences of inaccuracies can range from mild (e.g., the inconvenience of words being transcribed incorrectly) to catastrophic (e.g., incorrect transcripts resulting in inaccurate data which leads to a misdiagnosis).

To detect errors, improve accuracy and act as a fail-safe, we evaluated whether an automated syllable counting algorithm could be used alongside ASR. When compared to ASR, automated syllable counting algorithms are more reliable in the face of non-ideal recording conditions (Seshadri and Räsänen, 2019). We evaluated two questions of interest: (1) how much data are lost in the ASR transcription of speech recorded in non-ideal conditions, and (2) whether an automated syllable counter can approximate the true amount of speech (number of syllables uttered) and provide a way to estimate the quality of the transcription? We hypothesized a positive correlation between the proportion of syllables missing in a transcript (measured by a syllable counter) and the word error rate (WER) for that transcript (measured by the discrepancy between the ASR and human transcripts). If this proved correct, then the WER of a transcript should be possible to estimate using the proportion of missing syllables in a transcript. Transcripts from speech collected in sub-optimal recording conditions can thus be evaluated for suitability as a source of information about the speaker, even though the true WER is unknown.

## 2. Methods

### 2.1. Participants and procedures

Two different datasets were examined to illustrate the contrast between high- and low-quality audio recordings: (1) Telephone data - from a study assessing the cognitive state of older participants (Diaz-Asper et al., 2021). Participants (*N* = 91) were community dwelling, older English speakers (mean age = 73.7 ± 6.9 years of age) who had been called at home (on landlines, cellphones and speaker phones) and spoke for several minutes about past memorable experiences or hobbies. These data are a good test of ASR’s usefulness in applied settings, as they reflect a heterogeneous sample population with poor articulation and the recording quality was relatively poor (as reflected by the mean WER). (2) App data - from a project that collected speech recordings through a mobile device designed for ambulatory and longitudinal assessment of SMI, the “*delta* Mental State Examination” (Chandler et al., 2019; Holmlund et al., 2020). Participants included 25 patients (mean age = 49.7 ± 10.4 years of age) and 79 healthy controls, (mean age = 21.7 ± 1.4 years of age). Although a variety of speech data were collected, here we examined speech from a story recall task. This dataset contained much shorter recordings (average length 26 s across participant groups vs. 152 s in the Telephone data), of higher audio quality, although poor articulation and background noise was more prevalent in the patient samples. (See Table 1).

### 2.2. Analysis

Audio samples for each dataset were transcribed using both “gold standard” human transcribers and ASR (https://cloud.google.com/speech-to-text) so they could be compared and an accuracy score produced (specifically the WER was calculated for each speech sample by estimating the minimal edit distance with the Wagner-Fischer algorithm using the “jiwer” software package for Python (https://github.com/jitsi/asr-wer/). Only the participant’s speech was retained for the current analysis. For privacy preservation, each sample was checked for directly identifiable information and any such occurrences were removed.

Spoken syllables were automatically counted by analyzing a combination of audio intensity and voicedness to find syllable nuclei, using code from de Jong and Wempe (2009) for Praat software (Boersma and Weenink 2020, available here: https://sites.google.com/site/speechrate) with the Python package Parselmouth (https://github.com/YannickJadoul/Parselmouth). The count of spoken syllables for each recording was compared to the count of written syllables in the automated transcriptions (using the “syllapy” Python package; https://github.com/mholtzscher/syllapy), providing a ratio of the amount of syllables from the audio signal and the actual amount of information in the ASR transcriptions (the proportion of missing syllables).

Pearson correlations (derived with the “scipy” python package; https://scipy.org/) were used in the figures to illustrate strength of relationships between variables, with stronger correlations indicating a higher likelihood of detecting missing information in the transcripts. To evaluate the predictive performance of the method, linear regression models were built for each dataset and assessed with r^2^ and root mean squared error (RMSE) estimates across five-fold cross validation using the “scikit-learn” python package (https://scikit-learn.org/). We excluded recordings with extremely poor quality (*n* = 22, 3%), WERs over 100 (*n* = 6), proportion of missing syllables under −1 (*n* = 1) or word count in human transcription under 10 (*n* = 15). The latter exclusion cutoff was set to avoid very short responses where the proportion metric would become highly affected by just a few words missing in the automated transcriptions.

## 3. Results

### How much data are lost in machine transcription of speech recorded in applied settings?

**Fig. 1.**
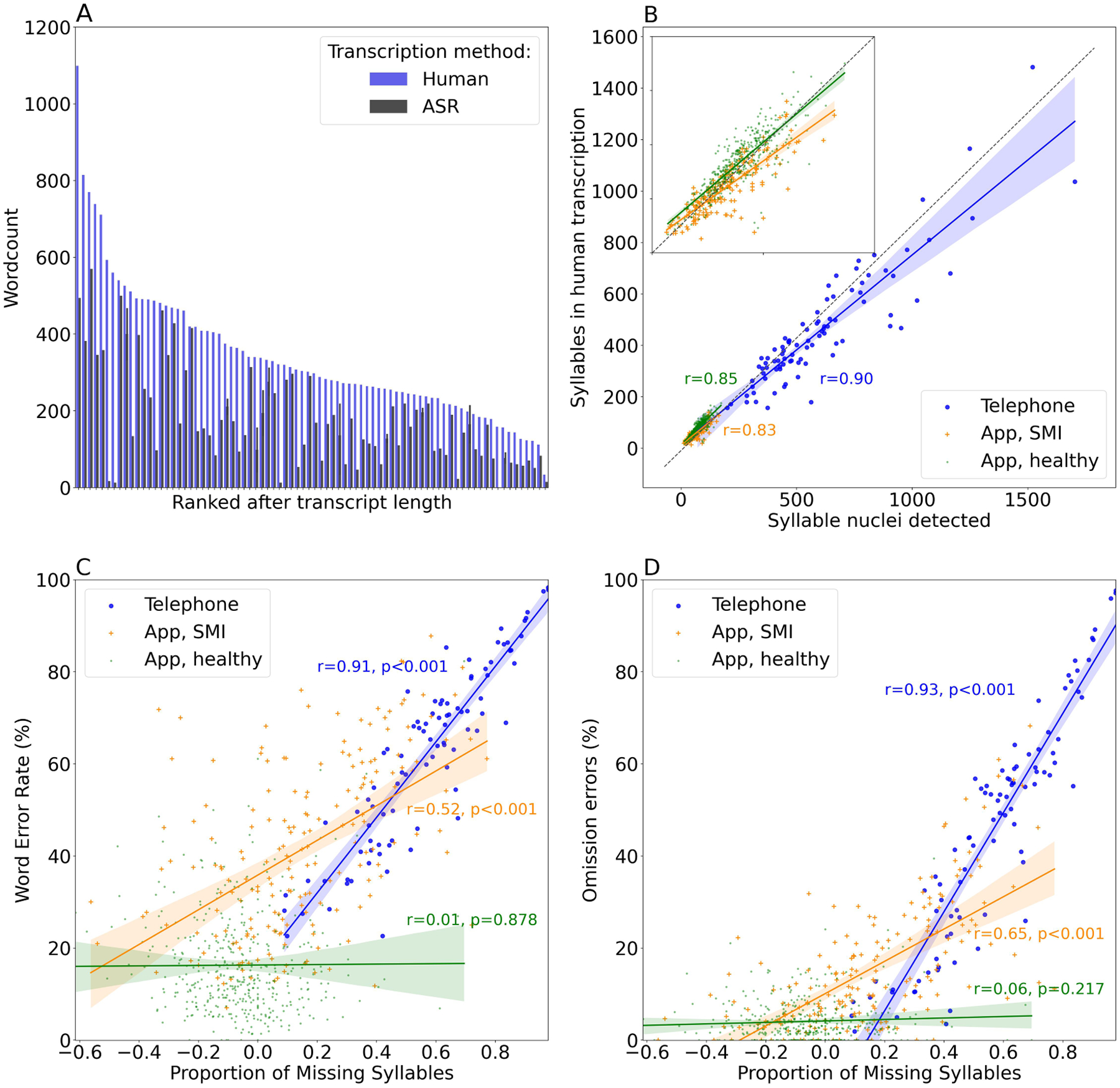
Panel A: An illustration of the sheer amount of information missing in some transcripts. Word count in human and ASR transcriptions of the samples collected in telephone interviews, ranked by length of transcript, shows how ASR transcriptions (black bars) had much lower word counts compared to the human transcriptions (blue bars). This was suggestive of a high incidence of omission errors. Panel B: Automatic syllable detection closely, but not perfectly, matched the amount of syllables detected and transcribed by humans. Panel C: The proportion of missing syllables in telephone interview transcripts (blue) showed a strong positive correlation with the word error rate (*r* = 0.91), supporting the notion that syllables missing can be used to estimate the amount of discrepancies in the ASR outputs from low quality recordings with challenging speaker characteristics. The relationship was weaker in the App samples from patients with SMI (orange, *r* = 0.52), and non-existent in high-quality recordings of healthy participants (green, *r* = 0.01). For figure with colors, please see online version. Panel D: Omission errors were likely to be the main source of missing syllables in transcripts and the relationship was indeed slightly stronger with omission errors specifically, rather than the overall WER.

For the Telephone samples, human transcriptions were substantially longer (mean = 337 words, SD = 167; Table 1 and Fig. 1, panel A) compared to machine transcriptions (mean = 180 words, SD = 127). Put differently, if one had relied on machine transcription, nearly half of the spoken words would be missed in a report of how much was said.

These omission errors resulted in high WER (mean = 63%, SD = 20%; Table 1). By contrast, in the higher quality App samples, the mismatch between the number of words in human and ASR transcriptions was not as apparent (mean WER across the two groups = 24%, SD = 18%), indicating that there was less information missing (i.e., fewer omission errors; Table 1), although there was a discrepancy in WER between the patient (mean WER = 45%, SD = 33%) and healthy control groups (mean WER = 17%, SD = 17%). (We note that state-of-the-art ASR systems typically produce WERs well under 5% on speech samples recorded in highly controlled conditions (El Hannani et al., 2021)).

### Can an automated syllable counter approximate the true amount of speech?

For the Telephone data, the approximate number of syllables was extracted from the raw audio signal and this number correlated strongly with the number of syllables in the human transcriptions (Fig. 1, Panel B). Differing amounts of missing information occurred across the range of transcript lengths but was most pronounced for the longer Telephone samples (Fig. 1, Panel C). The proportion of missing syllables showed a strong linear relationship with the WER (r(89) = 0.91, *p <* 0.001; r^2^ = 0.51–0.91; RMSE = 5.0 – 12.3; Fig. 1, Panel C), meaning that the quality of a transcription can be estimated by counting the number of sounds that are likely to be syllables. By contrast, in the App data, the correlation of the proportion of missing syllables and WER was substantially lower in the patients (r(192) = 0.52, *p <* 0.001; r^2^ = −0.12–0.47; RMSE = 12.1–18.6) and non-existent in the healthy controls (r(419)=0.01, *p* = 0.88; r^2^ = −0.05 to 0.01; RMSE: 9.1 – 13.3). The correlations between missing syllables and omission errors specifically indicated a stronger relationship (Telephone data: r(89) = 0.93, *p <* 0.001, r^2^ = 0.58–0.91, RMSE = 6.7–13.4); App data, patients: r(192)= 0.65, *p <* 0.001, r^2^ = 0.14–0.51, RMSE: 9.4–12.5; healthy: r(419) = 0.06, *p* = 0.22, r^2^ = −0.03–0.002, RMSE: 3.9–5.0; Fig. 1, Panel D), suggesting that it is possible to measure omission errors, but not substitutions or intrusions.

## 4. Discussion

Machine transcription metrics of speech quantity can be inaccurate as variable speaker characteristics and recording qualities contribute to substantial portions of speech being missed. We found that unconstrained speech samples from older people recorded in non-ideal conditions subjected to ASR resulted in transcriptions with unacceptably large error rates, notably omissions. By analyzing the audio signal (and not the transcription itself), an automated syllable counter was an effective method of quantifying the extent of missing information.

The method failed to generalize to a second speech dataset, likely reflecting the substantial differences between the two datasets; the App data were of high audio quality and were notably shorter than the Telephone data, and the topic of the speech was much more constrained. Although the patient group had a higher WER than healthy controls in the App dataset, the word errors were characterized primarily by substitutions, *not* omissions. Hence, the syllable counting method of estimating missing information appears most useful for longer samples of speech of poor audio quality, characterized by large amounts of background noise or participants with trouble enunciating. We do note however, that other characteristics of the speakers and recording mediums (such as dialect, cellphone versus landline etc.) could have contributed to the WER estimates beyond more obvious differences between the datasets (e.g., age and diagnostic group). Deciding when to look for evidence of missing information is currently done on a case-by-case basis (e.g., are the relevant speaker or recording characteristics present?), but future implementations may benefit from systematic estimation of audio quality (with e.g., Mittag et al. 2021). The implications of an inaccurate ASR transcription can range from inconvenient to disastrous; hence a fail-safe, such as the syllable counter described here, can be an important addition to commercial ASR systems when dealing with low quality audio recordings. The syllable counter can be considered agnostic to language, since it evaluates the error of the ASR system through the analysis of the audio signal itself instead of the written transcription. Importantly, this method can be applied to assess automated transcriptions independent of human involvement and as such can obviate issues with divulging personal private information by way of manual procedures.

## Figures and Tables

**Table 1:** Summary statistics of the two data sets.

	Telephone samples (*N* = 91, 91 samples)*		App samples (*N* = 104, 613 samples)^#^			
			Patients (*N* = 25, 193 samples)		Healthy (*N* = 79, 420 samples)	
	mean	std	mean	std	mean	std
Duration of samples, in seconds	152	67	29	14	25	8
Word counts, human transcriptions	337	169	45	21	61	21
Word counts, machine transcriptions	180	127	39	18	60	21
Word Error Rates (%)	63	20	45	33	17	17
- Omission errors (%)	47	25	15	14	4	5
- Substitution errors (%)	15	7	24	15	11	8
- Intrusion errors (%)	1	1	6	25	2	13
Syllable counts:						
a. From human transcriptions	453	229	58	27	81	28
b. From machine transcriptions	251	176	51	23	80	27
c. From counting syllable nuclei	597	280	64	30	76	27
Proportion of Missing Syllables (i.e., b/c)	0.57	0.22	0.16	0.26	−0.07	0.22

* From Diaz-Asper et al. (2021).

# From Holmlund et al. (2020).
